# Epigenetic Connections of the TRPA1 Ion Channel in Pain Transmission and Neurogenic Inflammation — a Therapeutic Perspective in Migraine?

**DOI:** 10.1007/s12035-023-03428-2

**Published:** 2023-06-16

**Authors:** Michal Fila, Elzbieta Pawlowska, Joanna Szczepanska, Janusz Blasiak

**Affiliations:** 1 Department of Developmental Neurology and Epileptology, Polish Mother’s Memorial Hospital Research Institute, 93-338 Lodz, Poland; 2 Department of Pediatric Dentistry, Medical University of Lodz, 92-217 Lodz, Poland; 3 Department of Molecular Genetics, University of Lodz, 90-236 Lodz, Poland

**Keywords:** Epigenetics, TRP channels, TRPA1, Pain transmission, Neurogenic inflammation, Migraine

## Abstract

Persistent reprogramming of epigenetic pattern leads to changes in gene expression observed in many neurological disorders. Transient receptor potential cation channel subfamily A member 1 (TRPA1), a member of the TRP channels superfamily, is activated by many migraine triggers and expressed in trigeminal neurons and brain regions that are important in migraine pathogenesis. TRP channels change noxious stimuli into pain signals with the involvement of epigenetic regulation. The expression of the TRPA1 encoding gene, *TRPA1*, is modulated in pain-related syndromes by epigenetic alterations, including DNA methylation, histone modifications, and effects of non-coding RNAs: micro RNAs (miRNAs), long non-coding RNAs, and circular RNAs. TRPA1 may change epigenetic profile of many pain-related genes as it may modify enzymes responsible for epigenetic modifications and expression of non-coding RNAs. TRPA1 may induce the release of calcitonin gene related peptide (CGRP), from trigeminal neurons and dural tissue. Therefore, epigenetic regulation of *TRPA1* may play a role in efficacy and safety of anti-migraine therapies targeting TRP channels and CGRP. TRPA1 is also involved in neurogenic inflammation, important in migraine pathogenesis. The fundamental role of TRPA1 in inflammatory pain transmission may be epigenetically regulated. In conclusion, epigenetic connections of *TRPA1* may play a role in efficacy and safety of anti-migraine therapy targeting TRP channels or CGRP and they should be further explored for efficient and safe antimigraine treatment. This narrative/perspective review presents information on the structure and functions of TRPA1 as well as role of its epigenetic connections in pain transmission and potential in migraine therapy.

## Introduction

Targeting calcitonin gene-related peptide (CGRP) and its receptor has opened a new era in the therapy of migraine, but it is still an undertreated disease, despite its high prevalence, serious physical and mental problems for affected individuals, and high burden for societies. One of the main reasons for that is incompletely known mechanisms of migraine pathogenesis and the action of anti-migraine drugs.

The importance of genetic component in migraine pathogenesis is reported in many association studies, in particular in familial hemiplegic migraines, but without a deeper look into the mechanisms underlying observed effects (reviewed in [1]). Functional studies suggest that mutations associated with migraine result in a worsened glutamatergic neurotransmission and cortical hyperexcitability, making the brain more susceptible to cortical spreading depolarization (CSD), a phenomenon important in migraine aura symptoms. Mutations in many functionally different genes associate with migraine and this reflects a genetic complexity of molecular pathophysiology of migraine. However, two groundbreaking papers showed an important role of DNA damage and repair in the regulatory processes in the central nervous system (CNS) [2, 3]. Although these discoveries were not reported in migraine cases, it may be concluded that DNA damage and repair may play a role in migraine pathogenesis [4]. Therefore, not only susceptibility variants, but also functional impairment in DNA metabolism may be essential in migraine pathogenesis. The relationship between genotype and phenotype, crucial for disease occurrence, is dictated by gene expression, which is principally regulated at the transcriptional and translational levels. Epigenetic regulation of gene expression, manifested by changes in DNA methylation, histone modifications, and action of non-coding RNAs, collectively creating the cellular epigenetic pattern, plays an important role in acquiring and maintaining normal or pathological brain phenotype (reviewed in [5]). Also, the expression of migraine-susceptibility variants of genes may result from disturbed epigenetic regulation of normal variants of genes important in migraine.

Epigenetics in diseases arouses an emerging interest as epigenetic regulation of gene expression can be modulated by environmental and lifestyle influences, including nutrition [6]. As some components of the diet are considered migraine triggers, epigenetics is postulated as a connection between diet and migraine [7]. This reflects a general tendency to consider the diet to modulate gene expression by changes in the epigenetic profile [8]. Although several kinds of “epigenetic diet” are regarded, many problems should be solved before implementation of such diets in migraine prevention as we showed for folate and DNA methylation [9]. However, epigenetic regulation of gene expression plays a role in migraine pathogenesis, as it plays an important role in every aspect of development of a phenotype, but the question “How?” is still open.

Regulation of the expression of the calcitonin related polypeptide alpha (*CALCA*) gene, encoding an isoform of CGRP and expressed in trigeminal ganglia, arouses a significant interest to improve migraine therapy targeting CGRP and its receptor. We recently provided some arguments that epigenetic regulation of the *CALCA* gene may be explored to increase efficacy and safety of migraine treatment [10]. Although targeting CGRP and its receptor with antibodies and antagonists was a breakthrough in migraine treatment, it is too early to assess the safety of long-term anti-CGRP treatment and these therapeutics might not be universal cure-all compounds as some patients do not show a satisfactory response [11, 12]. Stimulation of CGRP release by TRP channels suggests their involvement in neurogenic inflammation, an important aspect of migraine pathogenesis [13, 14].

Transient receptor potential (TRP) channels are expressed in trigeminal neurons and brain regions that are important in migraine pathogenesis and they change noxious stimuli into pain signals [15]. Their functional role in migraine was suggested in several studies and they are considered a migraine therapeutic target (reviewed in [16]). TRP channels are signaling molecules and defects in their genes are associated with many diseases, including hereditary disorders and TRP channelopathies [17]. Most of TRP channels are accessible for drugs as they are located on the cell surface. As TRP channels are multifunctional, they are challenging as a therapeutic target due to a need to reduce potential unwanted side effects. Some current antimigraine therapies, including that with botulinum neurotoxin type A in chronic migraine, affect TRP channels [18]. Moreover, TRP channels may affect CGRP release from trigeminal terminals [19]. Therefore, TRP channels present a potential in migraine therapy.

Epigenetics is dually associated with TRP channels as their genes and associated chromatin have a dynamic epigenetic profile [20]. On the other hand, TRP channels can also interact with enzymes responsible for the epigenetic pattern and control expression of some non-coding RNAs encoded by their introns [21, 22]. Therefore, epigenetics is an important aspect of the functioning of TRP channels.

Transient receptor potential cation channel subfamily A member 1 (TRPA1, TRP ankyrin 1), also known as the Wasabi receptor, is a member of TRP channels superfamily of ion channels. TRPA1, along with transient receptor potential cation channel subfamily V member 1 (TRPV1), TRPV4, and transient receptor potential cation channel subfamily M member 8 (TRPM8), are most consistently reported to associate with migraine among all TRP channels [23]. These channels respond to stimuli involved in migraine pathogenesis and TRPA1 is of a special interest in this regard as it is reported to be a link between stimulants of headache pain and migraine as well as mediate the vasodilatory response to environmental irritants evoking headache [24, 25].

Migraine as a channelopathy and an important role of epigenetic regulation in TRP channels in pain transmission justify studies on the potential role of epigenetics in TRP-targeting antimigraine drugs. Although there are many reviews on the TRPA1 channel, addressing mainly its role in pain transmission and inflammation, few of them deal with migraine, despite a mechanistic connection between TRP ion channels and CGRP signaling. Although epigenetic regulation of gene expression is an emerging subject in molecular neurobiology, epigenetic aspects of the control of TRPA1 gene are rarely addressed in TRPA1-related reviews and mainly focus on DNA methylation. In this narrative/perspective review, we present information on the role of epigenetics in TRPA1 channel regulation and its interaction with other proteins and regulatory RNAs in pain transmission, neurogenic inflammation, and pain-related syndromes with potential consequence for migraine pathogenesis. We also present arguments for the importance of epigenetic effects in a perspective TRPA1-targeting anti-migraine therapy.

## TRPA1 is a Member of Transient Receptor Potential Channel Gene Superfamily That Encode Multifunctional Cation Channels

### TRPA1 — the Gene and the Protein

Transient receptor potential cation channel subfamily A member 1 is a member of TRP channels superfamily of ion channels, consisting of 28 members in mammals. They are categorized into six main families: TRP ankrin (TRPA), TRP canonical (TRPC), TRP melastatin (TRPM), TRP mucolipins (TRPML), TRP polycystin (TRPP), and TRP vanilloid (TRPV). TRP channels are tetrameric forms of nonselective Ca^2+^ influx channels having six transmembrane domains with both termini embedded in the cytosol. They are involved in environment perception and sense various stimuli, including visual, gustatory, olfactive, auditive, mechanical, thermal, and osmotic influences. Most, if not all, of these stimuli can be, directly or indirectly, related to migraine. Mutations in TRP channels are associated with various diseases including neurological disorders and some of them have been classified as channelopathies (“TRPpathies”) [26].

TRPA1 is encoded by the *TRPA1* gene, which is located at 8q21.11, has 29 exons and 70,094 bp (GRCh38/hg38) and yields 3 transcripts translated to two 1119 aa and one 1144 aa proteins (https://www.ncbi.nlm.nih.gov/gene/8989, accessed January 19, 2023).

**Fig. 1:**
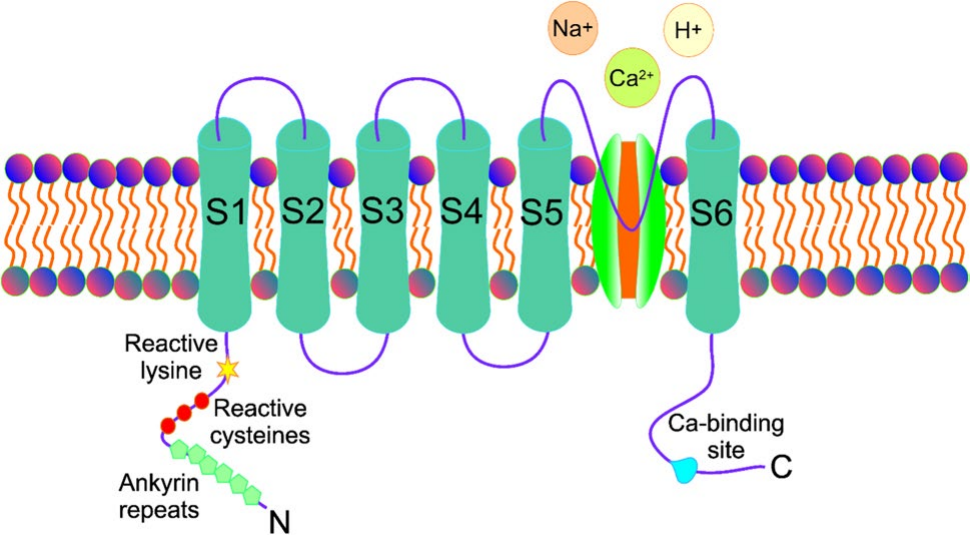
Structure of the transient receptor potential cation channel subfamily A member 1 (TRPA1) channel. TRPA1 contains 6 transmembrane domains (S1-S6) and its both termini (N and C) are embedded in the cytosol. The central pore region and selectivity filter for ions to enter and exit the cell membrane is located between S5 and S6. The N-terminus contains ankyrin repeats and reactive cysteine residues that are involved in many functions of TRPA1. The calcium binding site is localized in the C-terminus

TRPA1 belongs to the TRP ankyrin (TRPA) subfamily of the TRP channel superfamily. It is widely expressed in the cell surface of human cells, including neuronal cells [27]. TRPA1 is principally expressed on myelinated Aδ- and unmyelinated C-fibers of peripheral nerves. It can be found on cell bodies of dorsal root ganglion, nodose ganglia, and trigeminal ganglia neurons, as well as on the axons of spinal nerves, the vagus nerve, and trigeminal nerve [28]. TRPA1 is classified as a thermo-TRP as it can be gated by temperature changes and function as a temperature transducer [29]. The human TRPA1 has four subunits associating into a channel. Like remaining TRP channels, each TRPA1 subunit has six transmembrane domains, S1-S6, with bundles at the periphery of the channel formed by S1-S4 and a pore region and selectivity cation filter between S5 and S6 (Fig. 1). In its N-terminal region, TRPA1 has a domain of 14-18 ankyrin repeats, 33 aa each. This domain is important for the connection of TRPA1 with cytoskeleton, TRPA1 trafficking, and protein-protein interactions. However, N-terminal ankyrin repeats are not involved in the cold and chemosensitivity of the human TRPA1 [30]. The C621, C633, and C635 cysteines are essential for the TRPA1 response to reactive electrophiles and they are located in the membrane-proximal N-terminal region that surrounds the TRP-domain helix [31]. Channel opening is regulated by upper and lower gates formed by sidechains of asparagine 915 of pore helix 1 and hydrophobic S6 residues isoleucine 957 and valine 961, respectively, projected into the pore [31]. TRPA1 may also contain other, demonstrated and suggested, functional handles. The opening and closing of the gates are allosterically controlled by TRPA1 agonists and antagonists.

### TRPA1 in Pain Transmission and Neurogenic Inflammation

TRPA1 is activated by many reactive irritants, including cinnamaldehyde, allicin, allyl isothiocyanate, ligustilide, acrolein, and nicotine. TRPA1 activation in nociceptors evokes action potentials signaling pain and induces an aversive or protective response and malfunction of TRPA1 may result in impaired pain signaling [32].

**Fig. 2:**
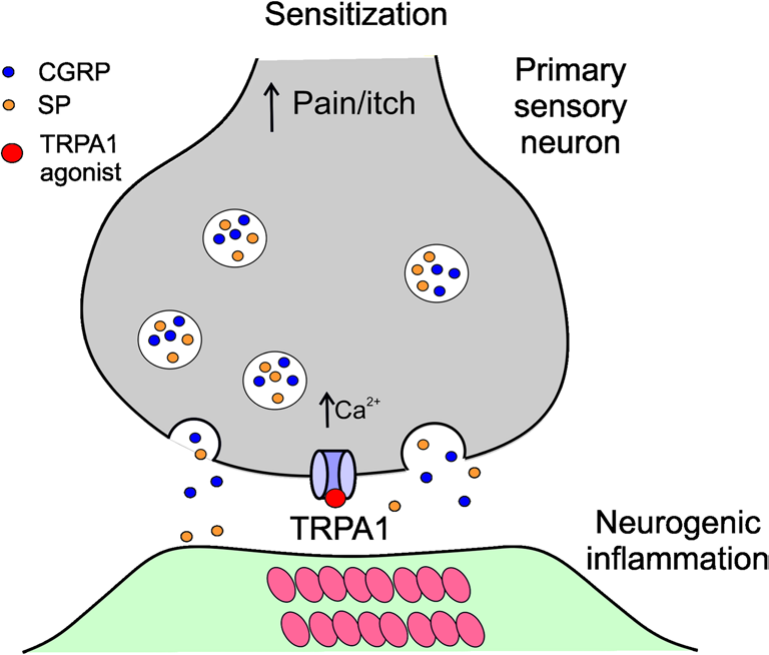
Involvement of the transient receptor potential cation channel subfamily A member 1 (TRPA1) channel in the pain signaling. A TRPA1 agonist may desensitize the channel that is expressed on primary sensory neurons. Activation of TRPA1 by agonists induces an increased calcium influx, the release of pro-inflammatory neuropeptides such as calcitonin gene-related peptide (CGRP) and substance P (SP), initiating the cascade of events resulting in neurogenic inflammation. At the same time, an impulse is generated that is sensed in the brain as pain or itching

TRPA1 agonists, including reactive oxygen and nitrogen species, support the N-methyl-D-aspartate (NMDA) receptor response and amplify pain signaling from primary sensory neurons expressing TRPA1 on their surface to projection neurons (Fig. 2) [32]. Activation of TRPA1 by agonists may lead to the release of neuropeptides, including CGRP and substance P (SP) that may generate neurogenic inflammation, an important aspect of migraine pathogenesis.

The activation of TRPA1 in rat dorsal root ganglion neurons increased its expression at mRNA and protein levels, as shown with the use of formalin and menthol that are TRPA1 agonist and antagonist, respectively [33]. TRPA1 activation induced an increased expression of CGRP, which was inhibited by pretreatment with PD98059, a mitogen-activated protein kinase 1/2 (MAPK1/2) inhibitor, but it did not affect TRPA1 expression in the presence of formaldehyde or menthol. It could be concluded from that study that TRPA1 agonists/antagonists upregulate/downregulate the expression of both TRPA1 and CGRP and the MAPK1/2 signaling pathway may mediate TRPA1-induced CGRP activation.

The involvement of TRPA1 in pain and inflammation may be partly underlined by endogenous TRPA1 agonists generated in oxidative stress or tissue injury, making it a druggable target in pain-related disorders [17, 34–36]. This is supported by many small molecular weight natural TRPA1 agonists and antagonists. A967079 and HC-030031 are pharmacological antagonists of TRPA1 shown to reduce peripheral neuropathy-associated pain and hypersensitivity in mice [37–40]. High-resolution examination of the TRPA1 structure revealed a ligand-binding pocket in a TRP channel that can be targeted by drugs [31].

As thermo-TRPs are expressed in C- and Aδ-fiber sensory neurons, which are involved in the transmission of pain signals, it was proposed that thermo-TRPs, including TRPA1, contributed to transmission and modulation of nociceptive signals [41]. This suggestion was supported by the observation that TRPA1 agonists induce a range of reactions from a pleasant hot feeling to unpleasant pain, as in the case of allyl isothiocyanate, a component of mustard or wasabi [42]. Another evidence of the involvement of thermo-TRPs in the control of pain transmission is a decrease in the intensity of some pain conditions by topical application of capsaicin, a selective agonist of TRPV1 [43]. TRPA1 stimulation was associated with an increased release of sensory neuropeptide from meninges and spinal dorsal root resulting in a neuroinflammatory state [28].

### TRPA1 in Migraine

Historically, it was TRPV1 and capsaicin that showed a potentially causative role of TRP channels in cluster headaches and migraine [44–46].

Many compounds, identified as TRPA1 agonists, including tobacco smoke, garlic, chlorine, formaldehyde, and others, are known as migraine attack triggers [47–51]. TRPA1 is plentifully expressed in primary sensory neurons and is considered sensors of chemical-, heat-, and mechanical-induced pain, playing a major role in migraine pain [15]. As mentioned, TRPA1 induces the release of CGRP.

There are some key discoveries relating TRPA1 to migraine pathogenesis. It was observed that exposure to extract from *Umbellularia californica* tree (“the Headache Tree”) provoked headache attacks [24]. Subsequently, it was shown that umbellulone stimulated TRPA1 and its stimulation in the dura by umbellulone and mustard oil induced headache responses in rats [52–54]. Moreover, umbellulone was shown to facilitate propagation of submaximal cortical spreading depolarization in mouse brain slices [55]. Umbellulone is an example of compounds extracted from natural products that desensitizes TRPA1 and meningeal nociceptors and presenting potential antimigraine effects [56, 57]. Obviously, umbellulone is not the only one environmental irritant that may be involved in migraine attack, so TRPA1 may be generalized as a mediator of the migraine-related effects of such substances.

The effect of umbellulone, along with another TRPA1 agonist, mustard oil, on the dural-projecting rat trigeminal ganglion neurons was studied with whole-cell patch-clamp recordings in vitro [52]. Application of both agonists to dural afferents produced TRPA1-like currents in about 40% of cells. Dural application of both agonists in in vivo behavioral model of migraine-related allodynia induced robust time-related tactile facial and hind paw allodynia that was weakened by pretreatment with the TRPA1 antagonist HC-030031. Furthermore, dural umbellulone and mustard oil decreased the number of vertical rearing episodes and the time spent rearing in comparison to vehicle-treated animals and this change in activity was prevented in rats pretreated with HC-030031 as well as sumatriptan, an antimigraine agent. This study showed that TRPA1 was expressed in a significant proportion of dural afferents, and activation of meningeal TRPA1 induced behaviors consistent with those observed in patients during migraine attacks. Furthermore, activation of meningeal TRPA1 could result in afferent signaling and headache. These results, although obtained in an animal model, support an important role of TRPA1 in migraine pathogenesis.

Nitroglycerine (NTG), a nitric oxide (NO) donor drug, is commonly used to induce migraine in experimental studies. NO was reported to target TRPA1 and nitrosylate its cysteine residues with a significant selectivity over other NO-sensitive TRP channels [36]. This process may lead to the channel sensitization and potentiation of CGRP release by other agents, resulting in increased neuroinflammation and pain response [41].

**Fig. 3:**
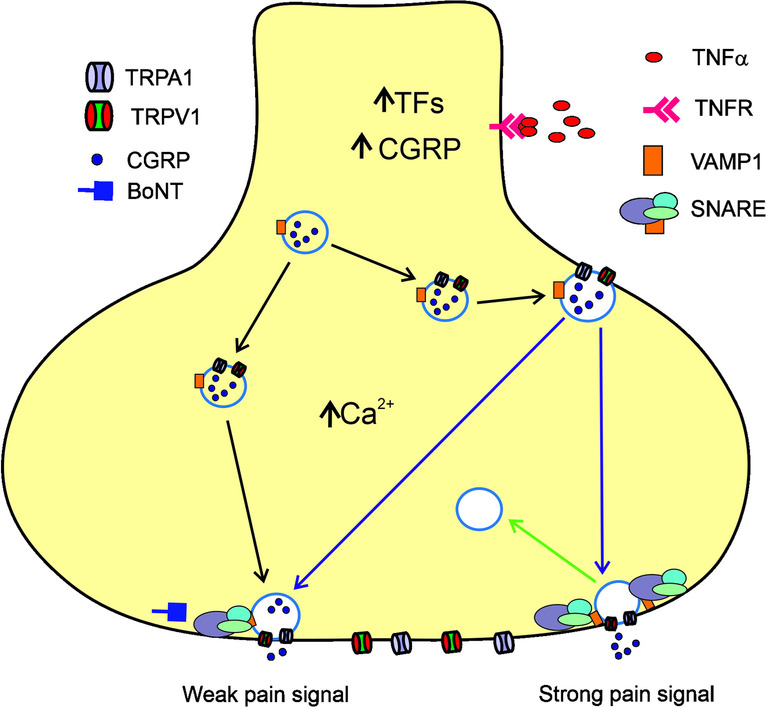
Tumor necrosis factor alpha (TNFα) induces pain-related effects in sensory neurons. TNFα is released by inflammatory cells during pain induction and binds its receptor (TNFR). This results in an increase in calcium ion concentration and the stimulation of several translational factors (TFs) to increase CGRP release, which is at a low level in normal conditions. These events lead to an enhanced trafficking of vehicles containing CGRP, transient receptor potential cation channel subfamily A member 1 (TRPA1), and transient receptor potential cation channel subfamily V member 1 (TRPV1) and eventually to release CGRP and other pain-mediators. TRPA1 and TRPV1 are loaded on vehicles containing CGRP and vesicle associated membrane protein 1 (VAMP1) and delivered to the plasma membrane with the formation of the SNARE complexes. Onabotulinum toxin-A (BoNTA) may affect the SNARE complex preventing exocytosis and delivery of TRPA1 and other TRP channels to the neuronal surface, resulting in a diminished CGRP release and pain intensity

Onabotulinum toxin-A (BoNTA) has been approved for the prevention of headache in chronic migraine and recent clinical evidence has shown that it is effective in the prevention of high-frequency episodic migraine [58, 59]. Benemei and Dussor pointed at the role of an interplay between BoNTA, CGRP, and TRP channels in migraine pathogenesis, concluding that BoNTA might decrease the release of CGRP due to TRP-related mechanisms [23]. Such mechanisms may include an association between CGRP release and the inflammatory response to tumor necrosis factor alpha (TNFα) (Fig. 3). It was shown that TRPA1 and TRPV1 were present on vesicles containing CGRP and colocalized on the fibers and cell bodies of cultured sensory neurons [60]. TNFα enhanced the surface content of both channels and induced their co-trafficking with the involvement of a synaptic vesicle associated membrane protein (VAMP1), essential for CGRP exocytosis. VAMP1 is component of the SNARE complex also containing synaptosome associated protein 25 (SNAP25), syntaxin 1A (STX1A), and syntaxin binding protein 1 (STXBP1). Furthermore, BoNTA may inhibit elevated delivery of TNFα. It can be speculated that similar processes may occur when only TRPA1 or another TRP channel is present in some exosomes [60].

ADM_12 is an antagonist of TRPA1, and it was shown to induce an anti-hyperalgesic effect in the second phase of the orofacial formalin test in rats bearing NTG-induced hyperalgesia at the trigeminal level [61]. This effect was associated with NTG-induced increase in TRPA1 mRNA indicating an active role of TRPA1 in reducing hyperalgesia at the trigeminal level and its importance in migraine pathogenesis. A reduction in the NTG-induced increase of the expression of the fos proto-oncogene, AP-1 transcription factor subunit (c-fos), a marker of neuronal activation following painful stimuli, important in migraine pathogenesis [62].

Activation and sensitization of the trigeminal primary afferent neurons innervating the dura and cerebral vessels is critical in migraine pathogenesis. It was shown that TRPA1-expressing neurons are clustered around a subset of dural afferent neurons, in contrary to TRPM8 channel [63]. It was concluded that TRPA1 channel contributed to the excitation of dural afferent neurons and the subsequent activation of the headache circuit, providing an anatomical basis for the functional significance of TRP channels in headache pathophysiology.

In a series of studies, Kunkler et al. showed that TRPA1 mediated meningeal vasodilation induced by environmental irritant that could induce sensitization of the trigeminal system and chronic migraine-like phenotypes in rats [64–66].

In summary, many migraine triggers are TRPA1 agonists. TRPA1 may be involved in the excitation of dural afferent neurons resulting in headaches. Also, TRPA1 may be important in the mechanisms of action of BoNTA, an effective drug in the prevention of chronic and high frequency, episodic migraine. NTG, a migraine-inducer, displays a high selectivity toward TRPA1 over other TRP channels.

## Epigenetic Connections of TRPA1 in Pain Transmission and Neurogenic Inflammation

Initially, epigenetics focused on dividing cells, but now an emerging interest is in studies on epigenetic processes within postmitotic cells, including these of the nervous system [67]. Several reports show contribution of epigenetic mechanisms in pain-related effects and their potential as a target in therapeutic strategies for acute and chronic pain (reviewed in [68]). Three main components of the cellular epigenetic profile are DNA methylation, postranslational histone modifications, and the action of non-coding RNAs. All these elements can be related to changes in the chromatin structure, affecting the expression of genes due to changes in accessibility of transcription factors and other members of gene expression machinery, but also some direct mechanisms are possible [69].

### DNA Methylation

Human DNA can be methylated at any site by chemicals that can transfer methyl group to DNA, but regulatory, signaling DNA methylation results from the transfer of a methyl group from S-adenosyl-methionine (SAM) to the carbon-5 of cytosine in DNA, catalyzed by the DNA methyltransferases DNMT1, DNMT3A, and DNMT3B [70]. Resulting 5-methyl cytosine (5mC) can be recognized and bound by specific proteins that change accessibility of transcription factors and other proteins as well as regulatory RNAs to gene promotors and other regulatory sequences. In the human genome, signaling DNA methylation occurs almost exclusively at cytosines located within the 5’-CpG-3’ (CpG) dinucleotides, which can be found in some locations within the human genome at frequency even tenfold higher on average. These clusters of CpG dinucleotides are called CpG islands. Methylated DNA may undergo passive or active demethylation [71]. Development of high-throughput technologies allows to study DNA methylation within the entire genome.

Pain sensitivity is a complex phenotype as it may be underlined by many biological, environmental/lifestyle, and psychological factors, but their effects depend on individual pain susceptibility, which is approximately only in half determined by genetic constitution. This conclusion is supported by twin-based heritability, animal research, and genetic association studies [72–76]. Pain-related signals are amplified in biological processes resulting in a chronic pain. These processes include sensitization of the nociceptive nerve fibers innervating peripheral tissues and sensitization of the spinal circuits relaying signals associated with tissue damage [77]. Again, these effects are only partly determined by genetic constitution and the role of epigenetic mechanisms in these processes is emerging [68, 78, 79].

Bell et al. examined genome-wide DNA methylation at 5.2 million loci by methylated DNA immunoprecipitation and deep sequencing in peripheral blood leukocytes of 50 identical twins and 50 unrelated individuals to identify differentially methylated loci associated with high or low heat pain sensitivity [77]. The most pronounced difference among nine differentially methylated DNA regions, showed the *TRPA1* gene. An increased expression level of *TRPA1* in the skin at higher pain thresholds was observed, in agreement with a downregulatory effect of DNA methylation in the *TRPA1* promoter.

A genome-wide methylation analysis on monozygotic twins showed that methylation of a CpG dinucleotide in the promoter of the *TRPA1* gene was inversely associated with the threshold for heat-induced pain [80]. It was found that the CpG dinucleotides that were associated with heat-evoked pain were hypermethylated in individuals with a low threshold for pressure pain. Females displayed a higher DNA methylation extent combined with higher pressure pain sensitivities, as compared with males. This study supports the role of epigenetic regulation of TRPA1 in pain sensitivity. Such role was confirmed in studies showing a positive correlation between DNA methylation levels in CpG island in the *TRPA1* gene in human peripheral blood leukocytes and a number of neuropathic pain syndromes, which, in turn, was negatively correlated with mRNA *TRPA1* expression [81]. In a similar study, an increased rate of DNA methylation at –51 CpG in the promoter of the *TRPA1* gene was positively correlated with the Douleur Neuropathique Questionnaire score in chronic pain patients [82].

Crohn’s disease is a type of chronic inflammatory bowel disease (IBD) associated with abdominal pain and involvement of sensory neurons innervating the gastrointestinal tract [83]. Activation of sensory neurons results in the release of neuropeptides, including CGRP, and individuals with IBD are reported to suffer from migraine at a higher rate than those without IBD [84, 85]. 2,4,6-trinitrobenzene-sulfonic-acid (TNBS), a TRPA1 agonist, induced IBD-like symptoms in mice associated with the release of CGRP and SP neuropeptides [86]. These data suggest that TRPA1 may mechanistically link IBD with migraine. Therefore, epigenetic regulation of TRPA1 in IBD may have some common pathways with its counterpart in migraine.

*TRPA1* was upregulated in colonic mucosa samples from Crohn’s disease patients [87]. Gombert et al. showed that such increased *TRPA1* expression in Crohn’s disease might be underlined by the disease-related changes in its epigenetic regulation [88]. They showed that the thresholds for pressure pain were lower in the patients than controls. Moreover, these thresholds were lower in females than males. Higher ratio of migraine in females is related to higher susceptibility to headache pain in women than men. Pain thresholds were negatively correlated with the CpG dinucleotide located 628 bp upstream from transcription start site in the promoter of the *TRPA1* gene. Again, that effect was more pronounced in female as compared to male patients. Furthermore, DNA methylation at the CpG in Crohn’s disease patients increased with age, whereas it decreased in controls. Pressure pain thresholds increased with age in both cohorts.

Multisomatoform disorder (MSD) is featured by distressing and functionally disabling somatic symptoms with chronic pain as the most frequent and clinically relevant complaint [89]. It was shown that DNA methylation at CpGs at –628 and –411 in the *TRPA1* gene in the blood cells was not correlated with mechanical pain threshold in MSD patients, but it showed a positive correlation with mechanical pain threshold in control subjects and female controls [90]. That study also showed a difference in DNA methylation levels between MSD patients with no and severe childhood trauma. DNA methylation of CpGs positively correlated with psychometric assessment of pain and pain levels rated on a visual analog scale. Therefore, that study confirms that DNA methylation or more general, epigenetic modifications within the *TRPA1* promoter, may be important in mechanical pain sensitivity. Epigenetic regulation of *TRPA1* may be influenced by childhood traumatic experiences in patients with MSD.

### Histone Modifications

The human genome is within a highly organized chromatin with histones as its major protein component. Histones undergo extensive post-translational modifications imposed mainly in their N-terminal tails, which protrude from the overall structure of histones and are accessible for enzymes covalently modifying their chemical structure by methylation, acetylation, phosphorylation, ubiquitination, and other alterations that collectively form a unique pattern of modifications (histone code). These modifications are read by specific proteins that are targeted by other proteins, remodeling the structure of chromatin and determining DNA accessibility for proteins and RNAs regulating gene expression.

The involvement of TRPA1 in neurogenic inflammation, important in migraine pathogenesis, may be linked with its functioning in immune cells. Macrophage polarization refers to activation and orientation of macrophages in response to a stimulus. It was shown that mice with a double knockout in the *TRPA1* and apolipoprotein E (*APOE*) genes showed an increase in atherosclerosis plaques compared to mice with single knockout in the *APOE* gene after a high-fat diet treatment [91]. Atherosclerosis was linked with a significant alteration of macrophage polarization toward a proinflammatory phenotype. Activation of TRPA1 by cinnamaldehyde decreased atherosclerosis progression. The effect on macrophage polarization was associated with a TRPA1-mediated induction of trimethylation of lysine 27 in H3 histone (H3K27me3), resulting in a downregulation of M1 macrophage genes. This epigenetic modification of H3 histone is regulated by the polycomb repressive complex 2 (PRC2), specifically through enhancer of zeste 2 polycomb repressive complex 2 subunit (EZH2). TRPA1 protected EZH2 from the proteasome-dependent degradation, allowing PCR2 to induce the H3K27me3 modification. Therefore, TRPA1 epigenetically regulated H3K27 trimethylation level in macrophages and so it can be considered both substrate for and inducer of epigenetic modifications.

High concentration of glucose in diabetes causes nerve damage and a vast majority of diabetic patients show diabetic neuropathy as a comorbidity and pain is the major symptom of this disorder [92]. Furthermore, although association between migraine and diabetes is not strongly established and requires further research, some data clearly support such an association [93]. In this context and the context of the role of epigenetic modifications of the *TRPA1* gene in pain transmission, the results obtained on the effect of histone acetylation on TRPA1 expression in diabetes may be important. Kong et al. showed that acetylation of H3 histone by histone acetyltransferase steroid receptor coactivator-1 (SRC1) at the *TRPA1* promoter might play a role in its expression and electrical activity of TRPA1 in diabetic rats [94]. That work focused on diabetes-related effects and did not provide details on the possible role of such epigenetic modifications in pain transmission. Histone acetylation at the gene promoter locus is usually associated with an increased expression of that gene, but such change should be considered in the context of whole set of epigenetic modifications that are imposed on that gene. Furthermore, functional changes in the product of this gene may not be related to the effects observed by Kang et al. and require further research, especially in pathological conditions (diabetes). In general, the involvement of TRPA1 in transmission of pain induced by common disorders, as cancer or diabetes and the role of epigenetics in this involvement is worth further studies as in many cases the most serious effects associated with a disease are these related to its secondary consequences. Type 2 diabetes mellitus is a good example of such state as its consequences, including neuro-, nephro-, and retinopathy result in more harmful effects than diabetes per se [95].

Wang et al. presented a mechanism leading from pressure overload to heart failure with the involvement of TRPA1 stimulating calcium/calmodulin dependent protein kinase II gamma (CAMK2G, CaMKII), which inhibits histone deacetylase 4 (HDAC4) [96]. HDAC4 prevents effective DNA binding by myocyte enhancer factor 2A (MEF2A, MEF2), contributing to hypertrophic gene expression and heart failure.

### miRNAs

Coding RNAs (mRNAs) constitute only a small (about 4%) fraction of the human transcriptome. The remaining 96% are called non-coding RNAs and can be classified into housekeeping ncRNAs and regulatory ncRNAs. The latter can be divided into short non-coding RNAs (snRNAs, less than 200 nucleotides) and long non-coding RNA (lncRNAs) (more than 200 nucleotides). Many species can be considered within these two categories and microRNAs (miRNAs) are the mostly explored species of regulatory ncRNAs. Several mechanisms underline involvement of regulatory ncRNAs in the control of gene expression, largely targeting mRNAs, but some ncRNAs interact with other regulatory RNAs and such an interaction is responsible for sponging effects, as a single ncRNA may have binding sites for several other regulatory RNA, preventing them from interaction with their target mRNAs. We did not find information on the role of long non-coding RNAs and circular RNAs in the regulation of TRPA1 so we have limited our considerations to miRNAs.

MicroRNAs mainly exert their role through the interaction with mRNA or other regulatory ncRNA. However, miRNAs can be found in circulation where they are stable and have been identified as useful biomarkers for many diseases [97]. Quereshi et al. showed changes in the miRNA profile in serum in several rodent models of pain, including spinal nerve ligation and spared nerve injury models of neuropathic pain; a complete Freund’s adjuvant (CFA) model of inflammatory pain; and a chemotherapy-induced model of pain [98]. Each model produced a unique miRNA expression profile, but with some common biological pathways in different models. All models showed alterations in the Wnt signaling pathway, which suggests that this pathway may be essential for pain pathogenesis. These studies showed usefulness of miRNAs in the circulation for pain-related studies and a perspective of their use in diagnosis and therapy of disorders associated with pain.

Unilateral spared nerve injury (SNI) model is used to investigate mechanisms of neuropathic pain induction [99]. It was shown that the expression of *Mus musculus* miR-449a (mmu-miR-449a) decreased in the SNI mice as compared with controls [100]. Transfection of dorsal root ganglion DRG neurons with that miRNA resulted in a decrease in *TRPA1* mRNA levels. This change occurred simultaneously with a decrease in mRNA calcium-activated potassium channel subunit α-1 (KCNMA1) and an increase in transmembrane phosphatase with tension homology (TPTE) in the DRG cells. However, a more detailed mechanism of possible interplay between TRPA1, KCNMA1, and TPTE mediated by mmu-miR-449a was not investigated and the only conclusion from that study was that mmu-miR-449a might decrease neuropathic pain through downregulation of TRPA1 and KCNMA1 and upregulation of TPTE. Consequently, mmu-miR-449a could be considered a potential therapeutic target in neuropathic pain treatment.

Neuropathic pain is one of the main side effects of several anticancer drugs, including cisplatin and its derivatives [101]. Clinically, chemotherapy-induced peripheral neuropathy involves deficits in sensory, motor, and autonomic functions underlined by several mechanisms, including those related to oxidative stress. Oxaliplatin, a cisplatin derivative, is a third-generation chemotherapeutic agent used to treat metastatic digestive tumors, but neuropathic pain as its side effects limits its application [102]. Intrathecal administration of miR-155 inhibitor weakened mechanical allodynia and cold hyperalgesia in rats treated with oxaliplatin, which upregulated TRPA1 [103]. This effect was associated with restoring of impaired expression of NFE2 like BZIP transcription factor 2-antioxidant response element (NFE2L2-ARE) in the dorsal horn. Furthermore, a diminished expression of NADPH oxidase 4 (NOX4) and consequently decreased levels of 8-oxoguanine (8-oxoG) were observed. Therefore, the effect of miRNA-155 on oxaliplatin-treated rats might be associated with increased oxidative stress. Inhibition of NOX4 decreased levels of products of oxidative stress in the dorsal horn and attenuated upregulation of TRPA1 induced by oxaliplatin. Therefore, TRPA1 may be important in a signaling pathway in which miR-155 modulates oxaliplatin-induced neuropathic pain via oxidative stress. Furthermore, epigenetic modifications of TRPA1 may be important for the action of miRNA-155 to ameliorate oxidative stress–related harmful side effects of oxaliplatin-based chemotherapy. Later studies confirmed an important, TRPA1-mediated role of miRNAs in modulating neuropathic pain induced by oxaliplatin. Zhang et al. elaborated a neuropathic pain rat model through intraperitoneal injection of oxaliplatin and measured mechanical withdrawal threshold and tail withdrawal latency [104]. Oxaliplatin downregulated the expression of miR-141-5p and upregulated TRPA1 at mRNA and protein levels in dorsal root ganglion. Intrathecal injection of miR-141-5p mimicked attenuated neuropathic pain induced by oxaliplatin and reduced the expression of TRPA1, a predicted target of miR-141-5p. It was concluded that TRPA1 might mediate miR-141-5p-relieved neuropathic pain induced by oxaliplatin.

Bortezomib is another chemotherapeutic agent inducing neuropathic pain as a side effect [105]. Using behavioral test to assess mechanical pain and cold sensitivity, it was shown that inhibition of miR-155 decreased mechanical allodynia and thermal hyperalgesia in the dorsal horn of the spinal cord of bortezomib-treated rats [106]. These effects were associated with downregulation of TRPA1 as well as pro-inflammatory tumor necrosis factor-α receptor and its downstream targets mitogen-activated protein kinase 14 (MAPK14, p38-MAPK) and mitogen-activated protein kinase 8 (MAPK8, JNK). Therefore, TRPA1 was involved in proinflammatory pathway mediated by TNRF and modulated by miR-155. As showed in the previous section, that pathway can be closely associated with pain-related effects. In fact, it was augmented by miRNA-155 mimics that amplified mechanical pain and cold sensitivity. Inhibition of TRPA1 and TNFR, p38-MAPK, and JNK decreased mechanical and thermal hypersensitivity induced by miRNA-155 mimics. Therefore, this is another work showing that epigenetic modification of TRPA1 by miRNA-155 may ameliorate neuropathic pain.

**Fig. 4:**
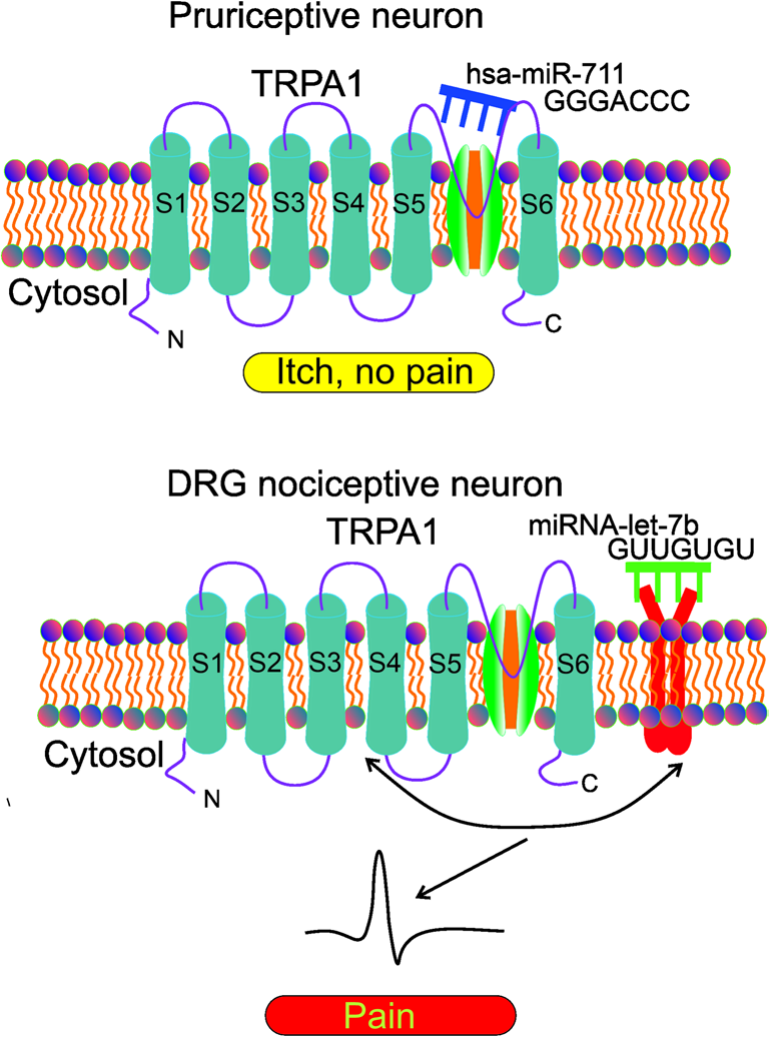
Role of microRNAs in the involvement of transient receptor potential cation channel subfamily A member 1 (TRPA1) in itch and pain dependent on and independent of toll-like receptor 7 (TLR7). In pruriceptive neurons, hsa-miR-711 binds TRPA1 in the extracellular S5-S6 loop with the core sequence 5’-GGGACCC-3’ driving chronic or acute itch, but no pain. In dorsal root ganglion (DRG) nociceptive neurons, miRNA-let-7b binds toll-like receptor 7 (TLR7) with its core sequence 5’-GUUGUGU-3’, but only in cells expressing TRPA1. The stimulation of the TLR7-TRPA1 interaction results in a fast inward current and action potential leading to pain

Although itch and pain are on different sensory pathways, TRPA1 may be involved not only in the activation of nociceptive neurons but also in their pruriceptive counterparts as shown by Han et al. [107]. They demonstrated that hsa-miR-711, secreted by inoculated human lymphoma cells on the mouse back skin, bound TRPA1 extracellularly on pruriceptive neurons. This binding involved the core 5’-GGGACCC-3’ sequence in the miRNA and the extracellular S5-S6 loop of TRPA1 to drive acute and chronic itch, but not pain (Fig. 4). In contrary, TRPA1 stimulation by allyl isothiocyanate, resulted in pain and not itch. It is puzzling how TRPA1 is involved in so different sensory pathways as pain and itch. It was hypothesized that itch was induced by fast and transient activation of TRPA1 by hsa-miR-711 from the extracellular side. Slow and persistent TRPA1 activation from the intracellular side by allyl isothiocyanate resulted in pain. The relationship between neuropathic pain and neuropathic itch is poorly known and neuropathic itch, similarly to migraine, involves the activation of the trigeminal system and rash was associated with migraine in some studies [108–110].

The work of Han et al. seems to be in a sharp contrast with the work of Park et al. who showed the role of extracellular miRNAs in a rapid excitation of nociceptor neurons via toll like receptor 7 (TLR7) and its coupling to TRPA1 ion channel [111]. They demonstrated that miRNA-let-7b induced fast inward currents and action potentials in DRG neurons. That effect required the 5’-GUUGUGU-3’ motif in miRNA-let-7 and occurred only in neurons that expressed TLR7 and TRPA1 and was abolished in mice with knockout in the *TLR7* or *TRPA1* gene. miRNA-let-7b induced TLR7/TRPA1-dependent single-channel activities in DRG neurons and HEK293 cells showing upregulated TLR7/TRPA1. Intraplanar injection of let-7b induced rapid pain via TLR7 and TRPA1. Lastly, miRNA-let-7b was released from DRG neurons by neuronal activation, and its inhibitor decreased formalin-induced TRPA1 currents and spontaneous pain. In conclusion, extracellular miRNAs may be pain mediators via TLR7/TRPA1 activation in nociceptor neurons. However, there is not any serious discrepancy between works of Han et al. and Park et al. The pain-unrelated effect observed by Han et al. was independent of TLR7 and included a direct binding to TRPA1, whereas Park et al. showed pain-related effects with the involvement of TLR7. Moreover, they used different miRNAs and the effect observed in either study cannot be generalized to all extracellular miRNAs, which was confirmed in the study of Han who showed that a mutated miRNA did not induce effects attributed to miRNA-711.

Winkler et al. suggested that TLR7-mediated divergent outcomes, such as neuronal apoptosis and pain stimulation in the central nervous system induced by extracellular miRNA-let-7b, might follow from different localization of TLR7 to the endosome in the cortical and hippocampal neurons or the plasma membrane in the sensory neurons [111–113]. Therefore, different types of neurons may traffic TLR7 to distinct locations in the plasma membrane, changing the functional response of neurons to stimulation by miRNA- let-7b. The general conclusion from both works is that extracellular miRNAs can regulate neuronal functions by interaction with TRPA1 that can be TLR7-dependent or independent. miRNAs were also found in DRG nociceptors to regulate pain and sodium channels expression [114].

The potential role of miRNAs in migraine pathogenesis was reported in several papers, but they presented mainly association studies, pointing at some miRNAs as migraine markers without a deep look into mechanisms underlying observed associations, despite bioinformatic identification of possible pathways involved into those associations. TRPA1 is a predicted target for many miRNAs (https://rgd.mcw.edu/rgdweb/genes/mirnaTargets.html?id=1303284&fmt=full), but functional studies linking these species with migraine are scarce.

## Conclusions and Perspectives

Epigenetic pattern plays an important role in the expression of all genes as DNA methylation and histone modifications determine the accessibility of a gene for transcription factors and other molecules controlling gene expression, including ncRNAs. Moreover, ncRNA may be activated or silenced through the direct interaction, first of all sponging effects, with other ncRNAs. Therefore, it is not surprising that epigenetic modifications of the *TRPA1* gene are reported in many pain-related conditions. Consequently, these modifications are justified to be considered to play a role in migraine pathogenesis and create a perspective in its prevention and therapy. Epigenetics is an emerging field in life sciences and the amount of data generated by epigenetic research is impressive, unraveling a crucial role of epigenetics in gene expression regulation. Epigenetic, cell-specific modulations affecting expression levels of ion channels are important for neuronal plasticity, adaptation, and homeostasis in neurons [115].

Although we did not cite direct evidence in migraine, epigenetic modifications of the *TRPA1* gene were shown to play an important role in the generation and transmission of neuropathic pain. Generally, migraine is not considered a neuropathic pain syndrome, but there are many common mechanisms and proteins involved in pathogenesis of migraine and neuropathic pain. One of them is TLRs and effects on the neuroimmune interface that justify considering a large extent of overlap between migraine and neuropathic pain [15, 116]. Allodynia, hyperalgesia, and expansion of nociceptive fields, associated with neuropathic pain, appear during most migraine attacks [117]. Migraine and neuropathic pain have some common core features: peripheral pain perception, peripheral sensitization at dorsal root ganglion or its cranial counterpart, including trigeminal ganglion and central sensitization at the spinal cord, brain stem nuclei and thalamus before definitive pain perception at the sensory cortical matrix [118].

Epigenetic modifications of TRPA1 were reported to play a role in other syndromes, including diabetes and diseases of the gastrointestinal tract that are reported to associate with migraine. Finally, epigenetic profile of the *TRPA1* gene as well as the interaction between circulating miRNA and *TRPA1* may be important in the release of CGRP, a key player in migraine pathogenesis.

The work on MSD as well as several other works relates pain transmission with the DNA methylation level in the promoter of the *TRPA1* gene in peripheral blood. Also, studies on Crohn’s disease concerned TRPA1 in colonic mucosa and not in enteric neurons. Therefore, there is a problem to justify conclusions on the effect in the nervous system based on results obtained in the periphery. Epigenetic pattern is largely perpetuated from one generation to another, but it is modified during development and stem cell differentiation and this modification can be tissue- or organ-specific. However, it is difficult, if not impossible, to study epigenetic modifications in migraine target tissues in live humans, not only due to ethical objections, but also for technical constraints. Moreover, animal models of neuropathic pain seem to be more consistent than their migraine counterparts and in consequence more reliable molecular data can be obtained in neuropathic pain studies [119].

In conclusion, there are many premises to study epigenetic connections in pain-related syndromes, including migraine. Such studies may open new and improve present therapeutic modalities in migraine.

## Data Availability

Not applicable

## Abbreviations

ADM1/2: Adrenomedullin 1/2
AMY: Amylin
AP-1: Transcription factor subunit
Aza-dC: 5-aza-2-deoxycytidine
bHLH-Zip: Basic HLH-leucine zipper
CALCA/B: Calcitonin related peptide alpha/beta
CCP: Katacalcin
CFA: Complete Freund’s adjuvant
CLR: Calcitonin-like receptor
CRCP: CGRP receptor component protein
CRE: cAMP-responsive element
CREB: cAMP response element binding protein
CXCL9: C-X-C motif chemokine ligand 9
DNMT1/3A/3B: DNA methyltransferase 1/3A/3B
DOCK6: Dedicator of cytokinesis 6
EZH2: Enhancer of zeste 2 polycomb repressive complex 2 subunit
CGRP: Calcitonin gene-related peptide
circRNA: Circular RNA
CSD: Cortical spreading depolarization
CT: Calcitonin
FOXA2: Forkhead box A2 protein
GWAS: Genome-wide association study
HDAC6: Histone deacetylase 6
HLH: Helix-loop-helix
lncRNA: Long non-coding RNA
KIF26A: Kinesin family member 26A
miRNA: Micro RNA
PM: Plasma membrane
NTG: Nitroglycerin
PTA: Posterior thalamic area
RAMP1: Receptor activity-modifying protein 1
RCP: Receptor component protein
RRE: Ras responsive element
SAM: S-adenosyl-methionine
SIRT1: Sirtuin 1
TCA: Trichostatin A
TGN: Trigeminal nerve
TNC: Trigeminal nucleus caudalis
TRPA1: Transient receptor potential cation channel subfamily A member 1
TSS: Transcription start site
USF: Upstream stimulatory factor
